# WKYMVm hexapeptide, a strong formyl peptide receptor 2 agonist, attenuates hyperoxia-induced lung injuries in newborn mice

**DOI:** 10.1038/s41598-019-43321-4

**Published:** 2019-05-02

**Authors:** Young Eun Kim, Won Soon Park, So Yoon Ahn, Dong Kyung Sung, Se In Sung, Jae Ho Kim, Yun Sil Chang

**Affiliations:** 1 Department of Health Sciences and Technology, Samsung Advanced Institute for Health Sciences and Technology (SAIHST), Sungkyunkwan University, Seoul, South Korea; 2 Department of Pediatrics, Samsung Medical Center, Sungkyunkwan University School of Medicine, Seoul, South Korea; 3 Samsung Biomedical Research Institute, Sungkyunkwan University School of Medicine, Seoul, South Korea; 4 Department of Physiology, School of Medicine, Pusan National University, Yangsan, South Korea

**Keywords:** Mechanisms of disease, Chronic obstructive pulmonary disease

## Abstract

The hexapeptide WKYMVm, which is a strong formyl peptide receptor (FPR) 2 agonist, exhibits pro-angiogenic, anti-inflammatory and anti-apoptotic properties. However, its therapeutic efficacy in bronchopulmonary dysplasia (BPD) has not been tested to date. Here, we investigated whether WKYMVm attenuates hyperoxia-induced lung inflammation and ensuing injuries by upregulating FPR2. The proliferation and tube formation ability of human umbilical vein endothelial cells (HUVECs), along with the level of extracellular signal regulated kinase (ERK) phosphorylation, were evaluated *in vitro*. Newborn mice were randomly exposed to 80% oxygen or room air for 14 days starting at birth. WKYMVm (2.5 mg/kg) was intraperitoneally administrated daily from postnatal day (P) 5 to P8. At P14, mice were sacrificed for histopathological and morphometric analyses. Along with upregulation of FPR2 and *p*-ERK, WKYMVm promoted HUVEC cell proliferation and tube formation *in vitro*. Additionally, WKYMVm promoted proliferation of human pulmonary microvascular endothelial cells (HULEC-5a) and murine pulmonary endothelial and epithelial cells *in vitro*. WKYMVm significantly attenuated hyperoxia-induced lung inflammation, as evidenced by increased inflammatory cytokines, neutrophils, and alveolar macrophages, and resultant lung injuries, which included impaired alveolarization and angiogenesis, an increased number of apoptotic cells, and reduced levels of growth factors *in vivo*, such as vascular endothelial growth factor and hepatocyte growth factor. WKYMVm attenuates hyperoxia-induced lung injuries and lung inflammation by upregulating FPR2 and *p*-ERK.

## Introduction

Despite recent advances in neonatal intensive care medicine, bronchopulmonary dysplasia (BPD), a chronic lung disease that occurs in premature infants receiving prolonged mechanical ventilation and oxygen supplementation, still remains a major cause of mortality and morbidity in survivors with few effective treatments^1,2^. Although BPD has a multifactorial aetiology, inflammation has been known to play a key role in the pathogenesis of BPD lung injuries including impaired alveolarization and angiogenesis^3,4^. Therefore, there is an urgent need to develop safe and effective anti-inflammatory agents as potential novel therapeutic candidates for BPD.

Recent studies have shown that the WKYMVm (Trp-Lys-Tyr-Met-Val-D-Met) hexapeptide, a strong formyl receptor (FPR) 2 agonist, has pleiotropic anti-inflammatory, pro-angiogenic, anti-apoptotic and immunomodulatory effects^5^ in various animal models of sepsis^6^, ulcerative colitis^7^, myocardial infarction^8^, ischemic hindlimb^9^ and diabetic cutaneous wound healing^10^. These data support the development of WKYMVm as a novel and effective anti-inflammatory therapeutic agent. However, the precise role of FPR2 in the pathogenesis of BPD and the functional significance of the FPR2 agonist WKYMVm in attenuating hyperoxia-induced neonatal lung injuries remain to be clarified.

Thus, in this study, we investigated the therapeutic efficacy of the FPR2 agonist WKYMVm in attenuating hyperoxia-induced lung inflammation and ensuing lung injuries, including impaired alveolarization and angiogenesis in newborn mice.

## Materials and Methods

### Isolation and culture of mouse lung endothelial cells

After 1- to 2-week-old mice (BALB/c) were anesthetized with ketamine/xylazine (140/14 mg/kg), ice-cold DMEM was injected via the right ventricle to flush the lungs of blood. One millilitre of collagenase type II (10 mg/ml) (GIBCO, Grand Island, NY) and DNase I (20 µg/ml) (Sigma-Aldrich, St. Louis, MO, USA) were quickly instilled through the trachea into the lungs, and then, the lungs were chopped as fine as possible. Chopped lungs were subsequently removed and incubated with 5 ml of collagenase II in a 50 ml tube for 30 min in a 37 °C shaking incubator. After the 40 min incubation, 25 ml of 1 × PBS was added to the tube. The tube was then vigorously shaken for 30 sec to dissolve the lung, and the resulting tissue/cell suspension was filtered through a 100 µm and a 40 µm strainer. Fetal bovine serum (FBS) was added to quench collagenase activity. The cells were centrifuged at 300 g for 10 min. The cells were washed once with 10 ml of HBSS/0.75% BSA and centrifuged again. After resuspension with 1 ml of sterile MACS buffer (PBS/0.75% BSA/2 mM EDTA), the cells were transferred to a new tube and centrifuged again at 400 g for 10 min. The cells were resuspended with 90 µl of MACS buffer and 10 µl of CD31-conjugated microbeads (Miltenyi Biotech, Bergish Gladbach, Germany). One millitre of MACS buffer was added to the cells, and the entire volume was applied to the column. The column was washed three times, and the cells were eluted. The cells were centrifuged at 400 g for 5 min and resuspended in 0.1% gelatin-coated plates. The purity of endothelial cells was determined with CD31 FACS analysis (Supplementary Fig. S2A).

### Isolation and culture of rat lung epithelial cells

After 4- to 8-week-old Sprague-Dawley rats were anesthetized with ketamine/xylazine (140/14 mg/kg), ice-cold DMEM was injected via the right ventricle to flush the lung of blood. A tracheal cannula was carefully inserted into the lung. We attached the barrel of a 1 ml syringe to the opening of the tracheal cannula and then slowly injected 1 ml of DMEM into the lung. We detached the syringe from the tracheal cannula and poured the lavage fluid from the lung. We repeated this procedure at least 6 times to remove as many macrophages as possible. The lungs were digested by instilling 2 ml of elastase (10 U/ml) at 37 °C and incubating for 20 min. The digested lungs were transferred to a Petri dish. After trimming away the trachea and major bronchi, the lung parenchyma was chopped using curved scissors into small, 1 to 2 cm^2^ pieces. Five millilitres of FBS was added to stop the digestion. Then, 15 mL of DMEM with 10 U elastase and 0.025% (w/v) DNase was added. The suspension was transferred to a 50 mL centrifuge tube and incubated in a water bath at 37 °C for 4 min. The cell suspension was filtered through a 100 µm and a 40 µm strainer. FBS was added to quench enzyme activity. The Percoll gradient was prepared in a sterile 50 mL centrifuge tube by layering 10 mL of light Percoll solution (1.040 g/mL) on top of 10 mL of heavy Percoll solution (1.089 g/mL). The preparation was centrifuged at 250 g for 20 min at 4 °C using a swingout rotor to produce a layer rich in alveolar type II cells at the interface between the Percoll gradients. Using a Pasteur pipet, the alveolar type II cell rich layer was transferred to a fresh centrifuge tube. The cells were washed by mixing them with 40 mL of ice-cold buffer (133 mM NaCl, 5.2 mM KCl, 1 mM NaH2PO4, 6 mM Na2HPO4, 10.3 mM HEPES, 5.6 mM glucose, pH 7.4) supplemented with 0.005% (w/v) DNase. The type II cells were pelleted by centrifugation (250 g for 20 min at 4 °C). The type II cell pellet was resuspended with 10 mL of cell culture medium and transferred to a culture dish. The purity of epithelial cells was determined with SP-C FACS analysis (Supplementary Fig. S2B).

### *In vitro* proliferation assay

The effect of WKYMVm on cell proliferation was investigated in the human umbilical vein endothelial cell line (HUVECs) (Invitrogen, Carlsbad, CA), human pulmonary microvascular endothelial cell line (HULEC-5a) (American Type Culture Collection, Manassas, VA, USA) and primary cultured murine lung endothelial and epithelial cells. For the ERK inhibition of proliferation assay in HUVECs, cells were exposed to an ERK-selective inhibitor (PD98059, 20 µM) (Sigma-Aldrich) for 4 hours before the WKYMVm (Anygen, Kwangju, Republic of Korea) treatment. In the hydrogen peroxide (H_2_O_2_)-induced oxidative stress in lung cell assay, cells were exposed to 100 µM H_2_O_2_ with WKYMVm treatment. After incubation with WKYMVm for 24 hours in 96-well plates, the cell counting kit (CCK)-8 (Dojindo, Kumamoto, Japan) assay was carried out to determine the relative cell proliferation rate (%), according to the manufacturer’s instructions.

### *In vitro* cell migration assay

The cells were grown to confluency in 12-well plates in culture medium containing 20 µg/ml mitomycin C (Sigma-Aldrich) for 4 h to completely inhibit cell proliferation. A straight scratch was made across the plate surface using a P200 pipette tip. The cells were then washed with PBS three times and further cultured in media with WKYMVm. After incubating for 0 and 24 h, the gap width reflecting re-population in the scratch was measured and recorded. This value was compared with the initial gap width at 0 h. Using ImageJ software (National Institute of Health, Bethesda, MD, USA), the size of the denuded area was determined at each time point from digital images.

### *In vitro* tube formation assay

For the endothelial tube formation assay to evaluate angiogenesis, 12-well plates were coated with Matrigel basement membrane matrix (Corning, Inc., Corning, NY, USA). Then 4 × 10^4^ HUVECs were seeded per well and incubated in culture medium with 0, 0.01, 1 or 100 µM WKYMVm. After incubation for 24 hours, the tube network was quantified by measuring tube length in pixels.

### FPR1 and FPR2 expressions *in vitro* and *in vivo*

The expression levels of FPR1 and FPR2 mRNA were measured by reverse transcription-PCR (RT-PCR). Total RNA was extracted with TRIzol, and then cDNA was synthesized using SMARTScribe™ Reverse Transcriptase (Clontech, Tokyo, Japan) with pd(N)6 random hexamers (Bioneer, Daejeon, Korea) according to the manufacturer’s instruction. PCR amplifications were performed with the following specific primers: human FPR2 forward primer 5′-CTGCTGGTGCTGCTGGCAAG-3′ and reverse primer 5′-AATATCCCTGACCCCATCCTCA-3′; human GAPDH forward primer 5′-TGCACCACCAACTGCTTA-3′ and reverse primer 5′-GGATGCAGGGATGATGTTC-3′; mouse FPR1 forward primer 5′-ACAGCCTGTACTTTCGAC-3′ and reverse primer 5′-CTGGAAGTTAGAGCCCGTTC-3′; mouse FPR2 forward primer 5′-ACAGCAGTTGTGGCTTCCTT-3′ and reverse primer 5′-CCTGGCCCATGAAAACATAG-3′ and mouse GAPDH forward primer 5′-ACCACAGTCCATGCCATCAC-3′ and reverse primer 5′-TCCACCACCCTGTTGCTGTA-3′. The PCR products were visualized with the E-Gel Power Snap Electrophoresis System (Invitrogen, Massachusetts, USA). Band intensities for each PCR product were measured using ImageJ software, and the FPR1/GAPDH and FPR2/GAPDH ratios were calculated. The protein level of FPR2 in lung tissue was measured by western blot. The membranes were blocked and incubated with the FPR2 primary antibody (1:1000; Novus Biologicals, Littleton, CO, USA) and then the appropriate secondary antibody (1:1000; DAKO, Glostrup, Denmark). The level of glyceraldehyde-3-phosphate dehydrogenase (GAPDH, 1:1000; sc-25778, Santa Cruz Biotechnology) was measured as a loading control. Protein signals were developed with ECL Prime Western blotting detection reagent (GE Healthcare, Piscataway, NJ, USA) and visualized on an Amersham Imager 600 (GE Healthcare). The FPR2/GAPDH ratio was calculated from the band intensities, measured using ImageJ software.

### Phosphorylated-extracellular signal regulated kinase signalling

To investigate whether extracellular signal regulated kinase (ERK) signalling is involved downstream of FPR2, the total and phosphorylated (p)-ERK protein levels were measured by western blot *in vitro* and *in vivo*. HUVECs or lung tissue were lysed using a protein extraction buffer (PRO-PREP solution; iNtRON Biotechnology, Inc., Seongnam, Korea), and the proteins were transferred to nitrocellulose membranes. The membranes were incubated with anti-total ERK 42/44 (1:2000; Cell Signaling Technology, Danvers, MA, USA) and anti-p-ERK 42/44 antibodies (1:2000; Cell Signaling Technology). Protein signals were developed with the ECL™ Prime western blotting detection reagent (GE Healthcare) and detected with an Amersham™ Imager 600. Detected band intensities were measured using ImageJ software, and the p-ERK/GAPDH ratio was calculated from the band intensities.

### Animal model of hyperoxia-induced lung injury

The experimental protocols were approved by the Animal Care and Use Committee of Samsung Biomedical Research Institute (Seoul, Korea). The procedures followed the institutional and National Institutes of Health guidelines for laboratory animal care, and animals were housed in an Assessment and Accreditation of Laboratory Animal Care International (AAALAC International)-accredited facility. Timed pregnant C57/BL6 wild type mice (National Experimental Animal Center, Pocheon, Korea) were housed in individual cages with free access to water and chow. Within 10 hours of birth, the newborn mouse pups were randomly assigned to one of four groups: normoxia control (NC), hyperoxic control (HC), normoxia with WKYMVm treatment (NWK) and hyperoxia with WKYMVm treatment (HWK). Gender was not considered during the treatment assignment, and all female and male mice were used in this study. Hyperoxic mice were raised in hyperoxic chambers (80% oxygen) for 14 days, while normoxic mice were raised in room air (21% oxygen). WKYMVm (2.5 mg/kg in 20 µl of normal saline), determined in an associated study^8^, or an equal volume of vehicle was administered intraperitoneally for 4 days from P5 to P8 according to the optimal therapeutic timing described in our previous study^11^. The mouse pups were kept at a constant temperature (24 °C) and humidity (50%) in a standard cage with a nursing mouse. Nursing mothers were rotated daily between litters in the normoxia and hyperoxia groups to avoid oxygen toxicity. We used 6 to 8 mouse pups per group for every read-out in histological and biochemical analysis. No mortality was observed during any animal experiment procedures.

### Tissue preparation

To harvest mouse lung tissue for histological evaluation, mice were sacrificed under deep pentobarbital anaesthesia (60 mg/kg, i.p.) at P14. After transcardiac perfusion with ice-cold normal saline, the lungs were inflated with normal saline and then immersed in 10% buffered formalin as described previously^11^. The fixed lungs were embedded in paraffin and sliced into 4 µm sections.

### Lung morphometry

Lung alveolarization was assessed using the mean linear intercept (MLI) and mean alveolar volume (MAV) on hematoxylin and eosin (H&E)- stained lung sections as described by Cooney and Thurlbeck^12^. The detailed method for measuring MLI and MAV has been described previously^11,13,14^.

### Measurement of medial wall thickness of pulmonary arteries

Pulmonary vascular remodeling was measured as the percentage of medial wall thickness (MWT) of small pulmonary arteries ((external diameter - internal diameter)/external diameter) x100%) according to a previous study^15^ using H&E-stained lung sections.

### Immunohistochemical analysis

The following primary antibodies were used as markers for type I and II alveolar epithelial cells, vascular endothelial cells, macrophages and neutrophils: aquaporin-5 (AQP5, 1:250; Abcam), pro surfactant protein C (SP-C, 1:2000; Millipore), Von Willebrand factor (vWF, 1:250; DAKO) and CD68 (1:250; Abcam), and myeloperoxidase (MPO, 1:50; Abcam), respectively. A FPR2 primary antibody (1:1000; Novus Biologicals) was used to immunohistochemically observe FPR2-expressing cells in lung tissue. Fluorescence microscope images were obtained using a confocal laser scanning microscope (LSM 700, Zeiss, Oberkochen, Germany). The light intensity of vWF-positive vessels was measured using ImageJ software (National Institutes of Health); we did not focus on the large blood vessels and instead assessed small- or medium- sized vessels for a proper lung angiogenesis assay. The numbers of CD68- and MPO-positive cells were counted in six non-overlapping fields by blind observers.

### Terminal deoxynucleotidyl transferase dUTP nick end labelling (TUNEL) assay

TUNEL staining was used to detect dead cells in lung sections using a DeadEnd Fluorometric TUNEL System kit (G3250; Promega, Madison, WI, USA) according to the manufacturer’s protocol. After nuclear counter staining with 4′,6-diamidine-2′-phenylindole dihydrochloride (DAPI), the number of TUNEL positive cells was counted in six non-overlapping fields under a confocal laser scanning microscope (LSM 700, Zeiss).

### Enzyme-linked immunosorbent assay

After HUVECs or mouse lung tissue were lysed in lysis buffer (R&D Systems, Minneapolis, MN, USA), each solubilized protein sample was adjusted to an equal concentration. The levels of inflammatory cytokines, namely interleukin 1α (IL)-1α and IL-6, and growth factors, such as vascular endothelial growth factor (VEGF) and hepatocyte growth factor (HGF), were measured using commercial ELISA kits (R&D System), according to the manufacturer’s protocol.

### Statistical analyses

Data are presented as the mean ± standard deviation (SD). One-way analysis of variance (ANOVA) followed by the Tukey-Kramer post hoc test was used for multiple comparisons. All statistical analyses were performed in GraphPad Prism 5 (GraphPad Software, Inc., San Diego, CA, USA). *P*-values less than 0.05 were considered statistically significant.

## Results

### Proangiogenic effect of WKYMVm in HUVECs

**Figure 1:**
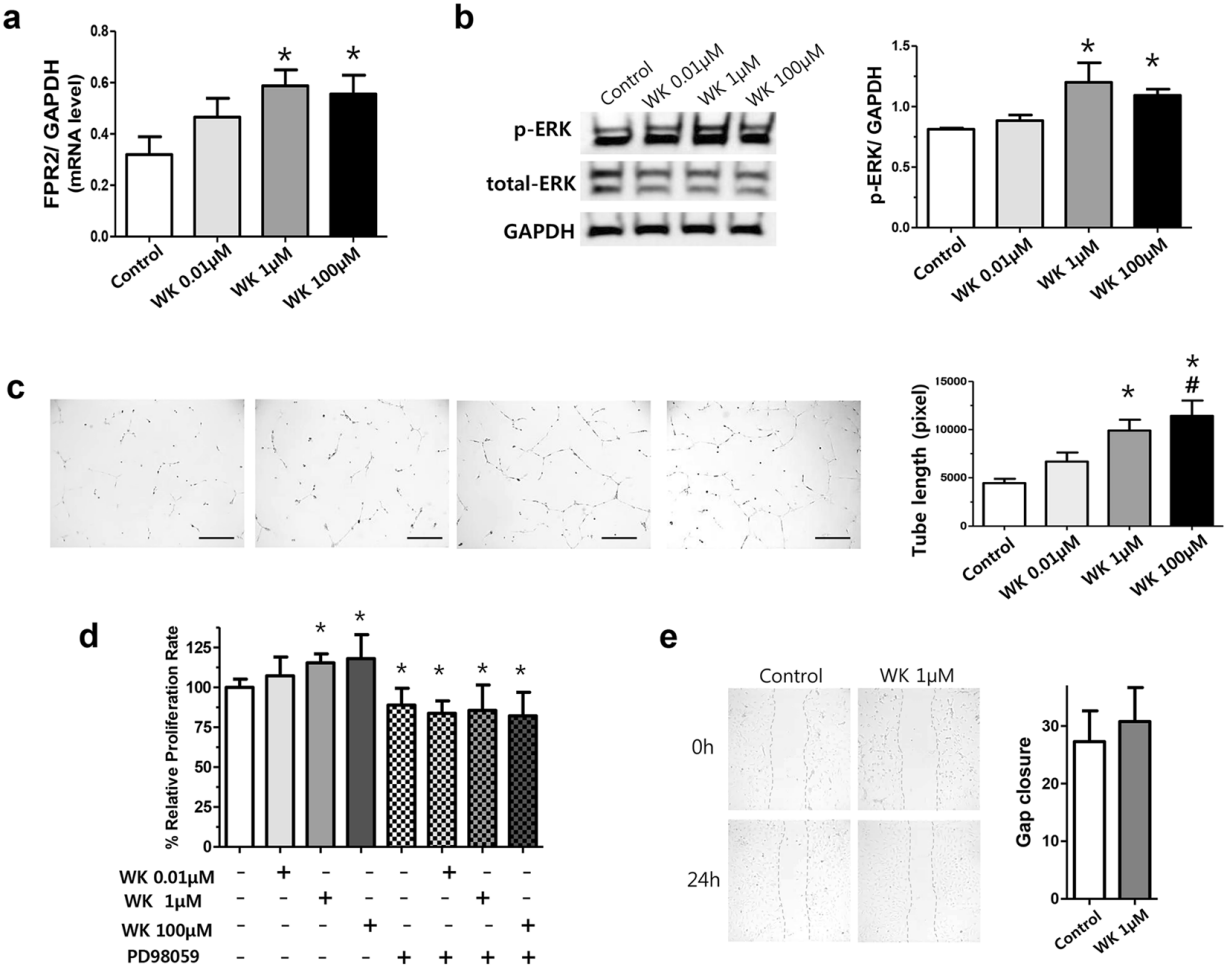
WKYMVm upregulated FPR2 and promoted angiogenic property in HUVECs. (**a**) mRNA level of FPR2, normalized to glyceraldehyde 3-phosphate dehydrogenase (GAPDH), measured using reverse transcription polymerase chain reaction (RT-PCR) in human umbilical vein endothelial cells (HUVECs). Full-length RT-PCR gels are shown in Supplementary Fig. S1A. (**b**) Representative western blots of total-ERK and phosphorylated (p)-ERK and its densitometric data, normalized to GAPDH, in HUVECs. Full-length Western blots are shown in Supplementary Fig. S1B. (**c**) Tube formation assay in HUVECs. Total tube length was measured in pixels. Images were taken at a magnification of 200 × (scale bar, 100 µm). (**d**) Cell proliferation, evaluated by CCK-8 assay, in HUVECs. ERK inhibitor, PD98059 (20 µM final), treated with WKYMVm. The results were expressed as relative proliferation rate (%) to treated control group. (**e**) Cell migration assay after scratching the monolayer of HUVECs. Quantitative data is provided by calculating the gap width of scratch re-population after incubating 24 hours compared with the initial gap size at 0 hour. Images were taken at a magnification of 100× (scale bar, 100 µm). Data are given as mean ± SD. *P < 0.05 vs. control group.

We used human umbilical vein endothelial cells (HUVECs), which are a gestational tissue-derived cell line obtained from an infant, to investigate the proangiogenic effect of WKYMVm in an *in vitro* assay. WKYMVm treatment at 1 and 100 µM, but not at 0.01 µM, significantly increased the FPR2 mRNA level (0.32 ± 0.22, 0.47 ± 0.21, 0.59 ± 0.21 and 0.56 ± 0.25 in the control, 0.01 µM, 1 µM and 100 µM WKYMVm groups, respectively; control vs 1 µM WKYMVm, *P* < 0.05; control vs 100 µM WKYMVm, *P* < 0.05); the treatment also increased the ERK phosphorylation level (0.81 ± 0.01, 0.88 ± 0.05, 1.20 ± 0.16 and 1.09 ± 0.05 in the control, 0.01 µM, 1 µM and 100 µM WKYMVm groups, respectively; control vs 1 µM WKYMVm, *P* < 0.05; control vs 100 µM WKYMVm, *P* < 0.05) compared to the control group (Fig. 1a,b). In the tube formation assay, the HUVEC total tube length was the greatest in the 100 µM WKYMVm condition, followed by 1 µM WKYMVm compared to the control group (4,437 ± 1,076, 6,671 ± 2,291, 9,896 ± 2,747 and 11,415 ± 3,905 in the control, 0.01 µM, 1 µM and 100 µM WKYMVm groups, respectively; control vs 1 µM, p < 0.05; control vs 100 µM, p < 0.05) (Fig. 1c). However, 0.01 µM WKYMVm-treated HUVECs did not have a significant improvement in tube length compared to the control group. In the cell proliferation assay, 1 and 100 µM WKYMVm, but not 0.01 µM of WKYVMm, significantly increased the percentage of relative cell proliferation compared to the control group (100.0 ± 15.48%, 107.2 ± 11.81%, 115.4 ± 5.53% and 118.0 ± 15.01% in the control, 0.01 µM, 1 µM and 100 µM WKYMVm groups, respectively; control vs 1 µM WKYMVm, *P* < 0.05; control vs 100 µM WKYMVm, *P* < 0.05) (Fig. 1d). To investigate whether the effect of WKYMVm on cell proliferation was ERK dependent, the WKYMVm was treated with exposure to an ERK inhibitor (PD98059) in HUVECs. In this experiment, none of the three dose of WKYMVm (0.01, 1 and 100 µM) combined with ERK inhibitor treatment significantly increased HUVEC proliferation compared to the control group (88.82 ± 10.59%, 83.71 ± 7.73%, 85.58 ± 15.9% and 82.10 ± 14.75% in the PD98059-treated control, 0.01 µM, 1 µM and 100 µM WKYMVm groups). However, in the cell migration assay, WKYMVm (1 µM) treatment did not significantly increase the cell migration rate relative to the control group (27.28 ± 5.32 and 30.75 ± 5.90 in the control and 1 µM WKYMVm groups, respectively) (Fig. 1e).

### Effect of WKYMVm on pulmonary endothelial and epithelial cell proliferation

**Figure 2:**
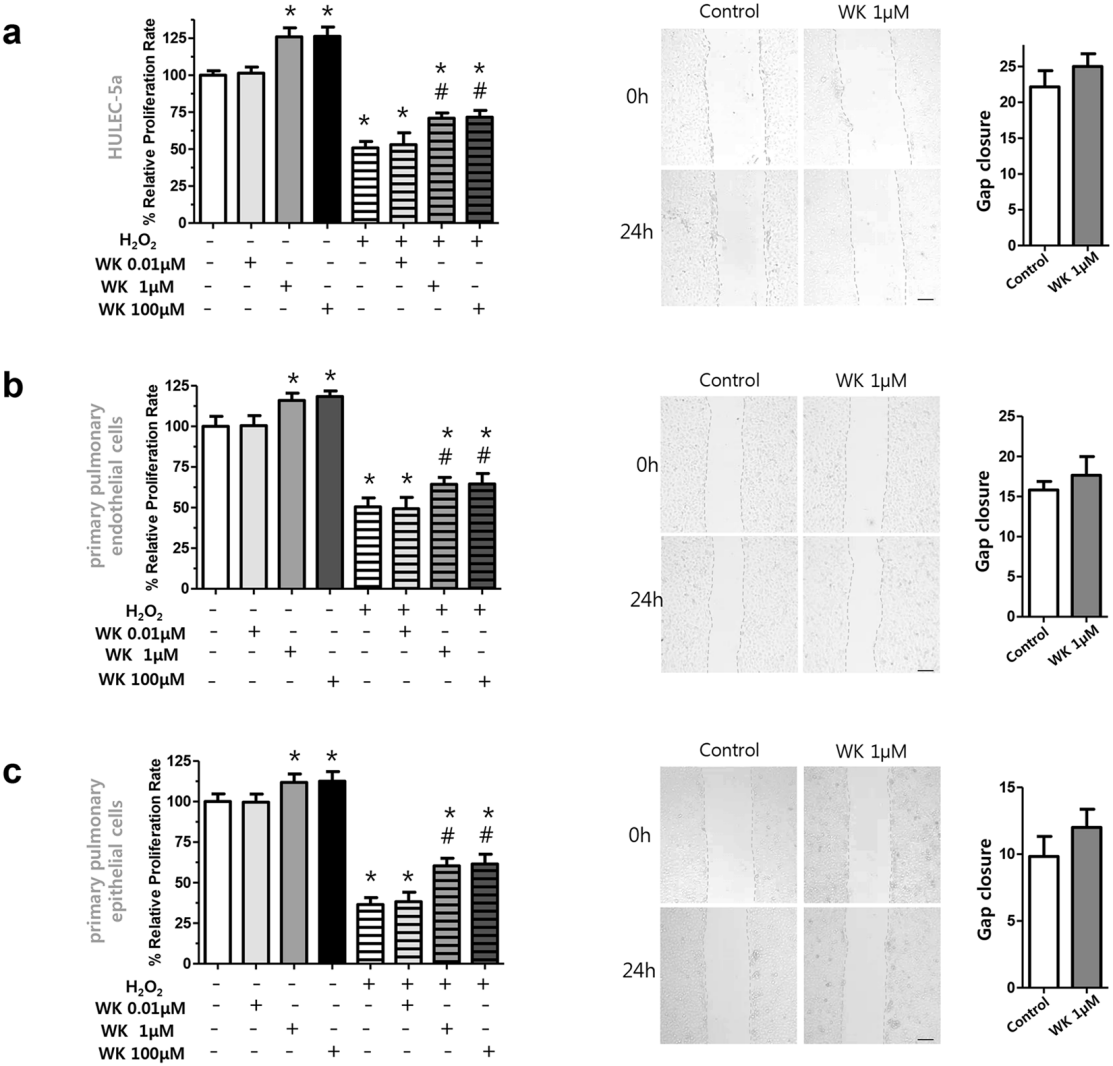
WKYMVm increased cell proliferation in pulmonary endothelial and epithelial cells. (**a**–**c**) Cell proliferation and cell migration assays in human pulmonary microvascular endothelial cell line (HULEC-5a), primary murine pulmonary endothelial and epithelial cells, respectively. Cell proliferation was evaluated by CCK-8 assay, and the results were expressed as relative proliferation rate (%) to control group. Cell migration was evaluated after scratching the monolayer of the cells. Quantitative data is provided by calculating the gap width of scratch re-population after incubating 24 hours compared with the initial gap size at 0 hour. Images were taken at a magnification of 100 × (scale bar, 100 µm). Data are given as mean ± SD. *P < 0.05 vs. control group. ^#^P < 0.05 vs. H_2_O_2_-exposed control group.

We also investigated whether WKYMVm affected proliferation in pulmonary endothelial and epithelial cells exposed to H_2_O_2_-induced oxidative stress. In human pulmonary microvascular endothelial cells (HULEC-5a) and primary murine pulmonary endothelial and epithelial cells, 1 and 100 µM WKYMVm treatments significantly increased proliferation in both the control (control vs 1 µM WKYMVm, *P* < 0.05; control vs 100 µM WKYMVm, *P* < 0.05, respectively) and H_2_O_2_-exposed groups (H_2_O_2_-control vs H_2_O_2_-1 µM WKYMVm, *P* < 0.05; H_2_O_2_-control vs H_2_O_2_-100 µM WKYMVm, *P* < 0.05, respectively) (Fig. 2a–c). However, WKYMVm (1 µM) treatment did not significantly increase the cell migration rate, in HULEC-5a and primary murine pulmonary endothelial and epithelial cells relative to the control group.

### FPR2 activation and ERK phosphorylation *in vivo*

**Figure 3:**
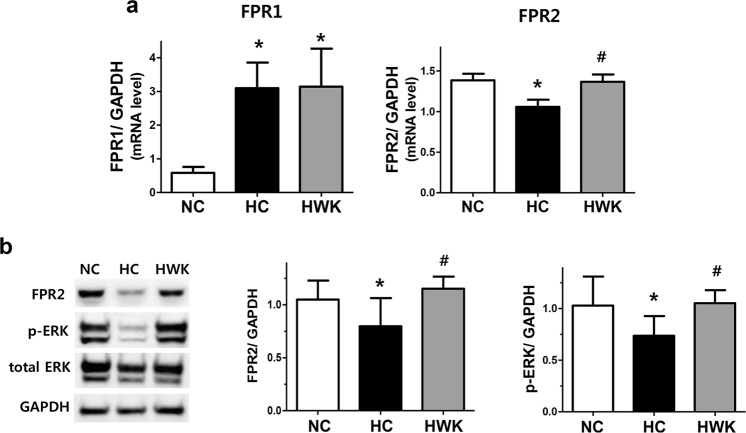
WKYMVm increased the expression of formyl peptide receptor 2 (FPR2) and phosphorylated extracellular regulated kinase (ERK) in the lungs of hyperoxic mice. (**a**) mRNA levels of FPR1 and FPR2, normalized to glyceraldehyde 3-phosphate dehydrogenase (GAPDH), measured using reverse transcription polymerase chain reaction (RT-PCR). Full-length RT-PCR gels are shown in Supplementary Fig. S3A. (**b**) Representative western blots of FPR2, total-ERK and phosphorylated (p)-ERK and its densitometric data, normalized to GAPDH. Full-length Western blots are shown in Supplementary Fig. S3B. Data are presented as mean ± SD. *P < 0.05 vs. normal control (NC). ^#^P < 0.05 vs. hyperoxia control (HC).

After hyperoxia-induced lung injury, the FPR1 mRNA level was significantly increased and the FPR2 mRNA level was significantly reduced compared to those in normoxic lungs (FPR1 mRNA level: 0.58 ± 0.18 and 3.09 ± 0.76 in NC and HC, respectively; NC vs HC, *P* < 0.001, FPR2 mRNA level: 1.39 ± 0.08 and 1.06 ± 0.09 in NC and HC, respectively; NC vs HC, *P* < 0.05) (Fig. 3a). The increased FPR1 mRNA level in hyperoxic lung was not significantly altered upon WKYMVm treatment. However, WKYMVm treatment significantly increased the levels of FPR2 mRNA (1.06 ± 0.09 and 1.37 ± 0.09 in HC and HWK, respectively; HC vs HWK, *P* < 0.05) and protein in HWK lungs compared to HC lungs (0.80 ± 0.26 and 1.15 ± 0.11 in HC and HWK, respectively; HC vs HWK, *P* < 0.05) (Fig. 3a,b). The phosphorylated (p)-ERK levels were significantly reduced by hyperoxia-induced lung injury compared to the normoxic control and significantly increased upon WKYMVm treatment (1.03 ± 0.28, 0.74 ± 0.19 and 1.05 ± 0.13 in NC, HC and HWK, respectively; NC vs HC, *P* < 0.05 and HC vs HWK, *P* < 0.05) (Fig. 3b). In the normoxic lung, WKYMVm did not significantly change the levels of FPR1, FPR2 and p-ERK (Supplementary Fig. S4).

### Lung histopathology

**Figure 4:**
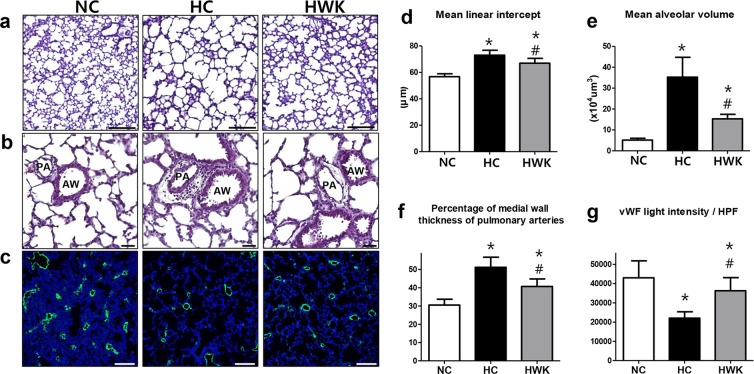
WKYMVm improved alveolarization, pulmonary vascular remodelling and angiogenesis in hyperoxic mice. (**a**,**b**) Representative photomicrographs of mice lungs stained with haematoxylin and eosin at a magnification of 200× and 400×, respectively (scale bar, 100 µm). (**d**,**e**) Morphometric evaluation of mean linear intercept (MLI) and mean alveolar volume (MAV), respectively. (**f**) Pulmonary vascular remodelling measured as a percentage of medial wall thickness. (**c** and **g**) Representative photomicrographs of von Willebrand factor (vWF, green) and its light intensity per high-power field (HPF), respectively. Cell counter-staining was performed using 4′,6-diamidine-2′-phenylindole dihydrochloride (DAPI, blue). Images were taken at a magnification of 100× (scale bar, 100 µm). Data are presented as mean ± SD. *P < 0.05 vs. normal control (NC). ^#^P < 0.05 vs. hyperoxia control (HC). PA = pulmonary artery. AW = airway.

The representative lung histology detected with a light microscope is shown in Fig. 4a. Compared to the small and uniform alveoli of the normoxic lung, there were fewer, larger and heterogeneous alveoli observed in the hyperoxic lung. These hyperoxia-induced impairments in alveolarization were attenuated by WKYMVm treatment. In the morphometric analyses, MLI and MAV, which respectively indicate the size and volume of alveoli, were significantly higher in the hyperoxic lung compared to the normoxic lung (MLI: 56.74 ± 2.22 and 73.02 ± 3.78 in NC and HC, respectively; NC vs HC, *P* < 0.001, MAV: 5.15 ± 0.86 and 35.26 ± 9.50 in NC and HC, respectively; NC vs HC, *P* < 0.001). These hyperoxia-induced increases in MLI and MAV significantly reduced upon WKYMVm administration (MLI: 73.02 ± 3.78 and 66.94 ± 3.69 in HC and HWK, respectively; HC vs HWK, *P* < 0.05, MAV: 35.26 ± 9.50 and 15.33 ± 2.14 in HC and HWK, respectively; HC vs HWK, *P* < 0.001) (Fig. 3d,e). In the pulmonary vascular remodelling analyses, we observed a significantly increased medial wall thickness in the small pulmonary arteries in the hyperoxic lung; however, this effect was significantly normalized after WKYMVm administration (30.50 ± 3.20, 51.14 ± 5.57 and 40.69 ± 4.09 in NC, HC and HWK, respectively; NC vs HC, *P* < 0.001 and HC vs HWK, *P* < 0.01) (Fig. 3b,f). The hyperoxic lung showed a significant reduction in vWF light intensity, which is indicative of impaired angiogenesis, compared to the normoxic lung (43,026 ± 8,789 and 22,043 ± 3,314 in NC and HC, respectively; NC vs HC, *P* < 0.001). However, WKYMVm administration significantly improved the impaired angiogenesis (22043 ± 3314 and 36281 ± 6835 in HC and HWK, respectively; HC vs HWK, *P* < 0.5) (Fig. 4b). In the normoxic lung, WKYMVm did not significantly alter MLI, MAV, medial wall thickness or vWF light intensity (Supplementary Fig. S5).

### TUNEL, CD68 and MPO staining

**Figure 5:**
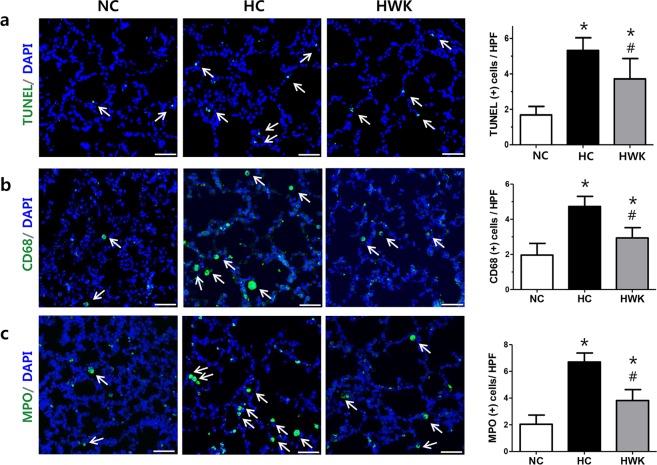
WKYMVm decreased the numbers of apoptotic and inflammatory cells in the lungs of hyperoxic mice. (**a**) Representative photomicrographs of terminal deoxynucleotidyl transferase dUTP nick end labelling (TUNEL, green) and its quantitative bar graph. (**b**) Representative photomicrographs of CD68 (green) and (**c**) myeloperoxidase (MPO) (green) with respective quantitative bar graphs. Cell counter-staining was performed with 4′,6-diamidine-2′-phenylindole dihydrochloride (DAPI, blue). Images were taken at a magnification of 200× (scale bar, 50 µm). Data are presented as mean ± SD. *P < 0.05 vs. normal control (NC). ^#^P < 0.05 vs. hyperoxia control (HC).

The number of TUNEL-positive apoptotic cells was significantly higher in the hyperoxic lung than in the normoxic lung; however, the hyperoxia-induced increase in TUNEL-positive cells was significantly attenuated by WKYMVm treatment (1.68 ± 0.48, 5.32 ± 0.72 and 3.71 ± 1.15 in NC, HC and HWK, respectively; NC vs HC, *P* < 0.001, NC vs HWK, *P* < 0.05 and HC vs HWK, *P* < 0.05) (Fig. 5a). The number of CD68-positive alveolar macrophages was significantly increased in the hyperoxic lung compared to the normoxic lung; however, the hyperoxia-induced increase in CD68-positive cells was significantly attenuated by WKYMVm treatment (1.96 ± 0.67, 4.73 ± 0.58 and 2.94 ± 0.58 in NC, HC and HWK, respectively; NC vs HC, *P* < 0.001, NC vs HWK, *P* < 0.05 and HC vs HWK, *P* < 0.001) (Fig. 5b). The number of MPO-positive neutrophils was significantly higher in the hyperoxic lung compared to the normoxic lung; however, the hyperoxia-induced increase in TUNEL-positive cells was significantly attenuated by WKYMVm treatment (2.04 ± 1.19, 6.70 ± 0.68 and 3.82 ± 0.82 in NC, HC and HWK, respectively; NC vs HC, *P* < 0.001, NC vs HWK, *P* < 0.05 and HC vs HWK, *P* < 0.001) (Fig. 5c). However, in the normoxic lung, WKYMVm did not significantly change the number of TUNEL-, CD68- and MPO-positive cells (Supplementary Fig. S6). In normoxic lung, WKYMVm did not significantly change the number of TUNEL-, CD68- and MPO-positive cells (Supplementary Fig. S7).

Through double staining with TUNEL and markers of endothelial cells (vWF) and type I and type II pulmonary epithelial cells (aquaporin 5 and pro surfactant protein-C, respectively), we found that most TUNEL-positive cells colocalized with pulmonary endothelial and epithelial cell markers.

### Levels of growth factors and inflammatory cytokines

**Figure 6:**
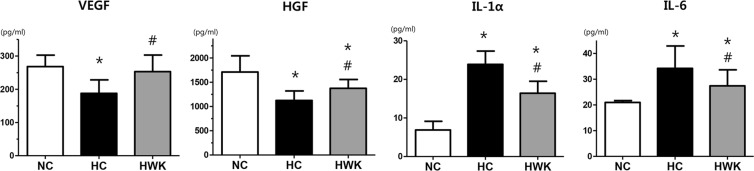
WKYMVm increased the levels of growth factors and decreased the levels of inflammatory cytokines. The levels of growth factors such as vascular endothelial growth factor (VEGF) and hepatocyte growth factor (HGF) and those of inflammatory cytokines such as interleukin-1α (IL-1α) and IL-6 measured by enzyme-linked immunosorbent assay (ELISA). Data are presented as mean ± SD. *P < 0.05 vs. normal control (NC). ^#^P < 0.05 vs. hyperoxia control (HC).

The levels of growth factors, such as vascular endothelial growth factor (VEGF) and hepatocyte growth factor (HGF), were significantly decreased in the hyperoxic lung; however, the VEGF and HGF levels were significantly improved after WKYMVm treatment (VEGF level: 268.2 ± 91.20, 187.7 ± 40.48 and 253 ± 50.01 in NC, HC and HWK, respectively; NC vs HC, *P* < 0.05 and HC vs HWK, *P* < 0.05, and HGF level: 1711 ± 333.6, 1124 ± 196.5 and 1376 ± 181.0 in NC, HC and HWK, respectively; NC vs HC, *P* < 0.05 and HC vs HWK, *P* < 0.05) (Fig. 6a,b).

The levels of the IL-1α and IL-6 inflammatory were significantly increased in the hyperoxic lung; however, the IL-1α and IL-6 levels were significantly reduced after WKYMVm treatment (IL-1α level: 6.88 ± 8.18, 23.89 and 16.42 ± 10.23 in NC, HC and HWK, respectively; NC vs HC, *P* < 0.01 and HC vs HWK, *P* < 0.05, and IL-6 level: 20.93 ± 0.72, 34.19 ± 8.68 and 27.41 ± 6.19, in NC, HC and HWK, respectively; NC vs HC, *P* < 0.01 and HC vs HWK, *P* < 0.05) (Fig. 6c,d).

## Discussion

Recent studies demonstrated that the WKYMVm hexapeptide, which is a strong FPR2 agonist, may be a promising peptide drug due to its pro-angiogenic and anti-inflammatory properties^16,17^. However, the role of FPR2 in BPD pathogenesis and the therapeutic efficacy of WKYMVm have not yet been delineated. In this study, WKYMVm significantly enhanced pro-angiogenic activity *in vitro* as evidenced by improved proliferation and tube formation in endothelial cells. Moreover, WKYMVm significantly attenuated the hyperoxia-induced increases in inflammatory responses as indicated by increased inflammatory cytokines, lung leukocytes, and alveolar macrophages; additionally, newborn mice treated with WKYMVm showed a significant improvement in lung injuries resulting from hyperoxia, including impaired alveolarization and angiogenesis, and increased TUNEL-positive cells. Our results are consistent with a previous report showing that WKYMVm treatment exerts protective effects against sepsis-induced death by enhancing the anti-microbial, anti-inflammatory and anti-apoptotic effects in a murine cecal ligation and puncture sepsis model^6^. WKYMVm has also been shown to inhibit apoptosis and stimulate neovascularization in a murine model of acute myocardial ischemia^8^, to induce neovascularization in a hind limb ischemia model^9^, and to have therapeutic effects on ulcerative colitis by inhibiting epithelial permeability and modulating the cytokine profiles^7^. Overall, these findings suggest that WKYMVm may be a potential novel and effective therapeutic agent for the management of neonatal hyperoxia-induced inflammation and ensuing lung injuries, i.e., BPD.

Although FPR1 is known to be a dominant pro-inflammatory formyl peptide receptor^18,19^, there was no significant increase in hyperoxia-induced FPR1 activity after WKYMVm treatment in this study. However, the hyperoxia-induced reduction in FPR2 activity was significantly improved upon WKYMVm treatment along with pro-angiogenic, anti-inflammatory, anti-apoptotic activities. These findings suggest that FPR2 has a critical role in hyperoxia-induced lung inflammation and ensuing lung injuries, highlighting that it may be a potential new therapeutic target in BPD. Moreover, *p-*ERK was significantly elevated along with upregulation of FPR2 after WKYMVm treatment both *in vitro* and *in vivo*. *In vitro*, WKYMVm treatment at 1 and 100 µM increased cell proliferation, but this effect was blocked when the ERK activation was inhibited. ERK phosphorylation is known to be involved in cell survival and angiogenesis^20^. Collectively, we can postulate that the beneficial effects of WKYMVm are secondary to ERK activation which suggests that the ERK signalling pathway is the downstream mediator for the pro-angiogenic, anti-inflammatory and anti-apoptotic activities of the FPR2 receptor agonist WKYMVm^20,21^.

In the present study, WKYMVm significantly attenuated the hyperoxia-induced reduction in the levels of growth factors such as VEGF and HGF. As these growth factors are essential for normal alveolar growth and angiogenesis^22,23^, their upregulation may contribute, at least in part, to the protective anti-inflammatory^24^, anti-apoptotic^25^ and pro-angiogenic effects^26^ of the WKYMVm peptide against BPD observed in this study. Although no direct interaction between FPR2 activation and growth factor secretion has been elucidated to date, FPR2 may indirectly upregulate the expressions of HGF or VEGF via an ERK signalling cascade to promote cell growth and survival^27^. Further studies will be necessary to dissect the intracellular signalling pathways triggered by WKYMVm, a strong FPR2 agonist, in the production of growth factors.

Determining the optimal dose of WKYMVm is an important and critical issue that needs to be addressed. Previous studies showed that a wide range of concentrations, spanning from 10 nM^16^ to 10 µM^9^, induced cell proliferation and angiogenesis *in vitro*. In this study, we tested a wider range of doses *in vitro* (0.01 µM to 100 µM) and found that a minimum of 1 µM WKYMVm was required to elicit angiogenic effects; however, no definite dose-response relationship was observed in HUVEC proliferation and tube formation with concentrations of up to 100 µM. We did not detect a significant increase in cell migration with WKYMVm treatment, suggesting that increasing cell proliferation rather than migration might be primarily responsible for the proangiogenic effects of WKYMVm.

WKYMVm is a simple synthetic hexapeptide (Trp-Lys-Tyr-Val-D-Met) with specific FPR2 agonist activity; therefore, WKYMVm can be easily manufactured at reduced production costs compared to recombinant proteins with complex structures. However, after injection, peptides might be rapidly eliminated from the blood through renal filtration^28^, and the therapeutic properties of injected peptides *in vivo* may be diminished by their rapid degradation. To overcome the low therapeutic efficacy of injected free peptides resulting from their short half-life *in vivo*, a large amount of the peptide (2.5–8 mg/kg/day) was repeatedly injected 2–4 times in this and other studies^28^. Several strategies have been developed to prolong the peptide half-life, such as chemical modification, liposomal encapsulation, and polymeric encapsulation to enhance the *in vivo* stability and biological activity and, consequently, reduce the dose and frequency of injection^9,28–31^. Therefore, further studies are required to better define the optimal dosing strategy for WKYMVm.

In the present study, we did not determine the distinct mechanism by which the WKYMVm increases FPR2 expression in the hyperoxic lung. We postulate two possible ways. First, WKYMVm might directly increase the FPR2 promoter activity in treated cells. Second, WKYMVm might increase the number of FPR2-expressing cells by preserving pulmonary endothelial and epithelial cells through inhibition of apoptosis and promotion of angiogenesis in the hyperoxic lung. In the lung, FPR2 is expressed in bronchial epithelial cells, pulmonary endothelial cells and immune cells, according to references^32–34^. We observed that FPR2 is expressed in pulmonary endothelial and epithelial cells and macrophages, as evidenced by immunostaining with aquaporin-5, pro surfactant protein C and CD68, respectively, in this experiment (Supplementary Fig. S8). Because we did not measure the number of cells expressing FPR2 or its magnitude of expression after treatment, further studies are needed to clarify these points. In the present study, a bronchoalveolar lavage fluid cell count would further support the inflammation data, but we were technically unable to lavage in this study because of the small-sized (average 6 g) 14-day-old newborn mice. Moreover, we could not measure the levels of MPO and other pro-inflammatory cytokines using ELISA, due to the very small sample size of lung tissue obtained from each newborn mouse. Therefore, we only measured IL-1α and IL-6, which are well known pro-inflammatory cytokines that are elevated in chronic lung diseases in preterm infants^35^. Because various other molecular mediators of angiogenesis, such as cytokines and intracellular signalling pathways^36^, may be involved, they should be investigated in future studies.

In summary, WKYMVm, a synthetic hexapeptide with strong FPR2 agonist activity, showed pro-angiogenic activity *in vitro*, and protected against hyperoxia-induced lung inflammation and resultant lung injuries such as impaired alveolarization and angiogenesis and increased apoptosis. Our results showed two main therapeutic strategies that promote angiogenesis and attenuate inflammation in hyperoxia-induced lung injury in newborn mice. Our findings suggest that activation of FPR2 is important for treating hyperoxia-induced lung injury and that WKYMVm may be a promising BPD treatment.

## Supplementary information

supplementary material
