# Supplementary material for: WKYMVm hexapeptide, a strong formyl peptide receptor 2 agonist, attenuates hyperoxia-induced lung injuries in newborn mice
Source: Sci Rep. 2019 May 2;9:6815. doi: 10.1038/s41598-019-43321-4 (PMC6497690; doi:10.1038/s41598-019-43321-4)

Supplementary material

WKYMVm hexapeptide, a strong formyl peptide receptor 2 agonist, attenuates hyperoxia-induced lung injuries in newborn mice

Young Eun Kim[1¶], Won Soon Park[2, 3¶], So Yoon Ahn[2], Dong Kyung Sung[3], Se In Sung[2], Jae Ho Kim[4], Yun Sil Chang[1, 2, 3*]

[1]Department of Health Sciences and Technology, Samsung Advanced Institute for Health Sciences and Technology (SAIHST), Sungkyunkwan University, Seoul, Korea, [2]Department of Pediatrics, Samsung Medical Center, Seoul, Korea, [3]Samsung Biomedical Research Institute, Sungkyunkwan University School of Medicine, Seoul, Korea, [4]Department of Physiology, School of Medicine, Pusan National University, Yangsan, Korea

¶These authors contributed equally to this work.

*Corresponding Author: Yun Sil Chang, MD, PhD.

Department of Pediatrics, Samsung Medical Center, Sungkyunkwan University School of Medicine, 50 Irwon-dong, Gangnam-gu, Seoul 135-710, Korea

Tel: +82.2-3410-3528; Fax: +82.2-3410-0049

E-mail: yschang@skku.edu

Supplemental Figure Legends.

Figure S1. Full-length RT-PCR gels shown in Figure 1A and full-length Western blots shown in Figure 1B.

Figure S2. The purities of murine pulmonary endothelial cells and epithelial cells were determined with CD31 and SP-C, respectively, in FACS analysis

Figure S3. Full-length RT-PCR gels shown in Figure 3A and full-length Western blots shown in Figure 3B

Figure S4. WKYMVm did not significantly affect the expression levels of formyl peptide receptor (FPR) 1, FPR2 and phosphorylated (p)-ERK in the lungs under normoxic condition. (A) mRNA levels of FPR1 and FPR2, normalized to glyceraldehyde 3-phosphate dehydrogenase (GAPDH), measured using reverse transcription polymerase chain reaction (RT-PCR). (B) Protein levels of FPR2 and p-ERK, normalized to GAPDH, measured using western blot. Data are presented as mean ± SD.

Figure S5. WKYMVm did not significantly affect alveolarization, pulmonary vascular remodelling and angiogenesis in the lungs under normoxic condition. (A and B) Representative photomicrographs of mice lungs stained with haematoxylin and eosin at a magnification of 200× and 400×, respectively (scale bar, 100 μm). (D and E) Morphometric evaluation of mean linear intercept (MLI) and mean alveolar volume (MAV), respectively. (F) Pulmonary vascular remodelling measured as a percentage of medial wall thickness. (C and G) Representative photomicrographs of von Willebrand factor (vWF, green) and its light intensity per high-power field (HPF), respectively. Cell counter-staining was performed using 4′,6-diamidine-2′-phenylindole dihydrochloride (DAPI, blue). Images were taken at a magnification of 100× (scale bar, 100 μm). Data are presented as mean ± SD.

Figure S6. WKYMVm did not significantly affect the numbers of TUNEL-, CD68 and MPO-positive cells in the lungs under normoxic condition. Representative photomicrographs of TUNEL-, CD68- and myeloperoxidase (MPO)-positive cells (green; upper, middle and lower panels, respectively) with the respective quantitative bar graphs. Cell counter-staining was performed with DAPI (blue). Images were taken at a magnification of 200× (scale bar, 50 μm). Data are presented as mean ± SD.

Figure S7. WKYMVm did not significantly affect the levels of growth factors, such as VEGF and HGF, and inflammatory cytokines, such as IL-1α and IL-6, in the lungs under normoxic condition. Data are presented as mean ± SD.

Figure S8. Double-immunostaining of FPR2 with the markers of each pulmonary cells, such as vWF (a marker of vascular endothelial cells), aquaporin-5 (AQP5) (a marker of type I pulmonary epithelial cells), pro-surfactant protein-C (SP-C) (a marker of type II pulmonary epithelial cells) and CD68 (a marker of alveolar macrophages)

Figure S9. Double-staining of TUNEL-positive cells with the markers of each pulmonary cells, such as vWF (a marker of vascular endothelial cells), AQP5 (a marker of type I pulmonary epithelial cells) and SP-C (a marker of type II pulmonary epithelial cells)

Figure S1

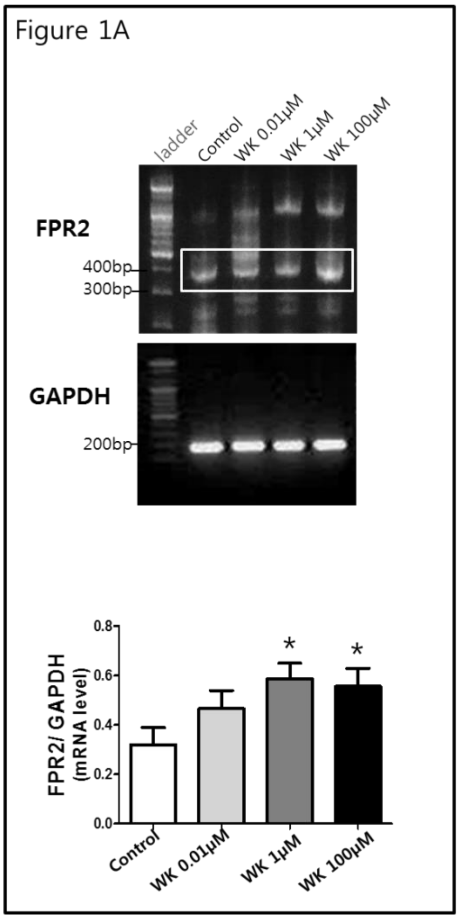

Figure 1A
ladder
Control
WK 0.01μM
WK 1μM
WK 100μM
FPR2
400bp
300bp
GAPDH
200bp
FPR2/ GAPDH (mRNA level)
0.8
0.6
0.4
0.2
0.0
Control
WK 0.01μM
WK 1μM
WK 100μM

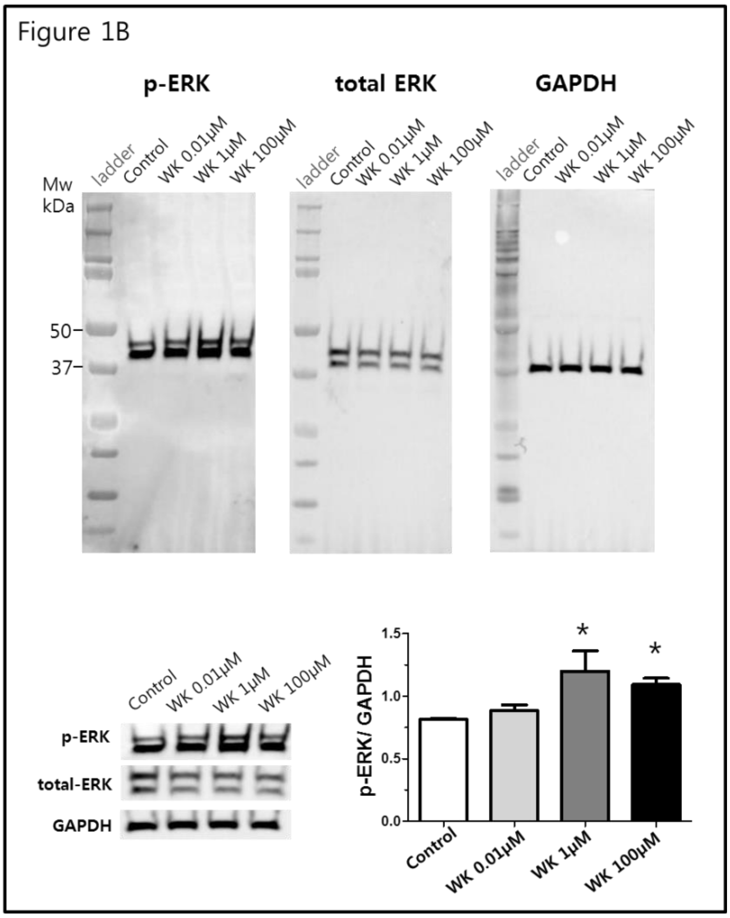

Figure 1B
p-ERK
total ERK
GAPDH
ladder
Control
WK 0.01μM
WK 1μM
WK 100μM
Mw kDa
50
37
p-ERK
total-ERK
GAPDH
p-ERK/ GAPDH
1.5
1.0
0.5
0.0
Control
WK 0.01μM
WK 1μM
WK 100μM

Figure S2

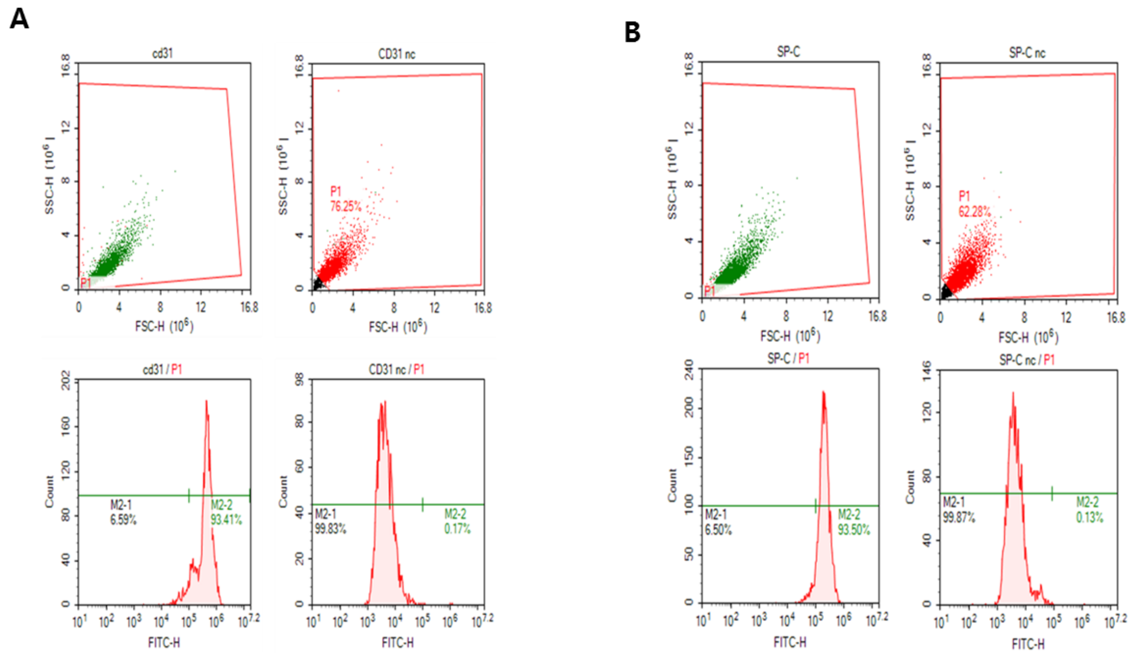
A
B
cd31
CD31 nc
SP-C
SP-C nc
P1
P1 76.25%
P1 62.28%
cd31 / P1
CD31 nc / P1
SP-C / P1
SP-C nc / P1
M2-1 6.59%
M2-2 93.41%
M2-1 99.83%
M2-2 0.17%
M2-1 6.50%
M2-2 93.50%
M2-1 99.87%
M2-2 0.13%
SSC-H (10⁶)
FSC-H (10⁶)
Count
FITC-H

Figure S3

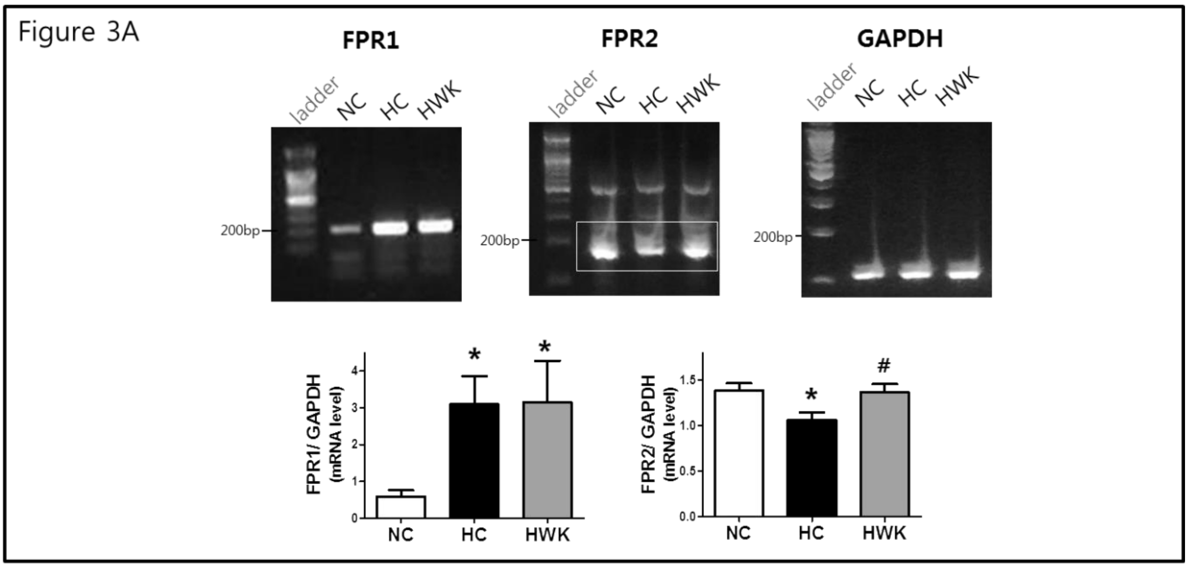
Figure 3A
FPR1
FPR2
GAPDH
ladder
NC
HC
HWK
200bp
FPR1/ GAPDH (mRNA level)
FPR2/ GAPDH (mRNA level)
*
#

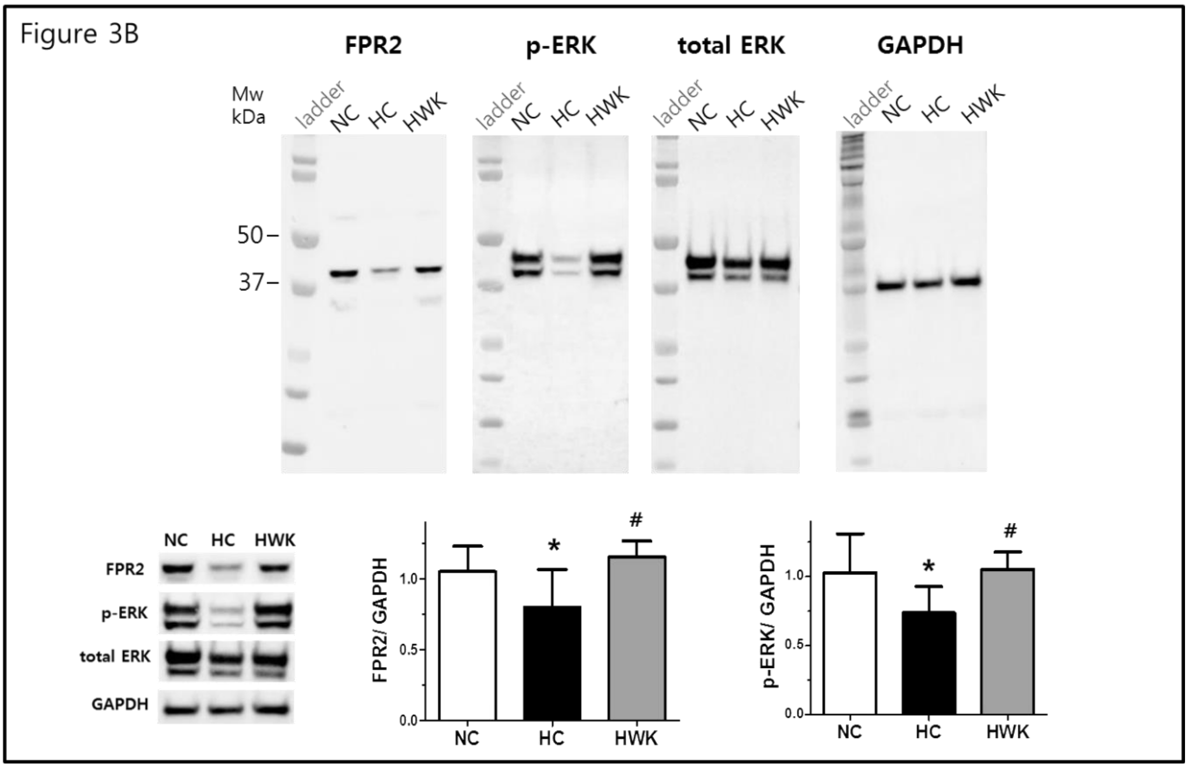
Figure 3B
FPR2
p-ERK
total ERK
GAPDH
Mw kDa
ladder
NC
HC
HWK
50
37
FPR2/ GAPDH
p-ERK/ GAPDH
*
#

Figure S4

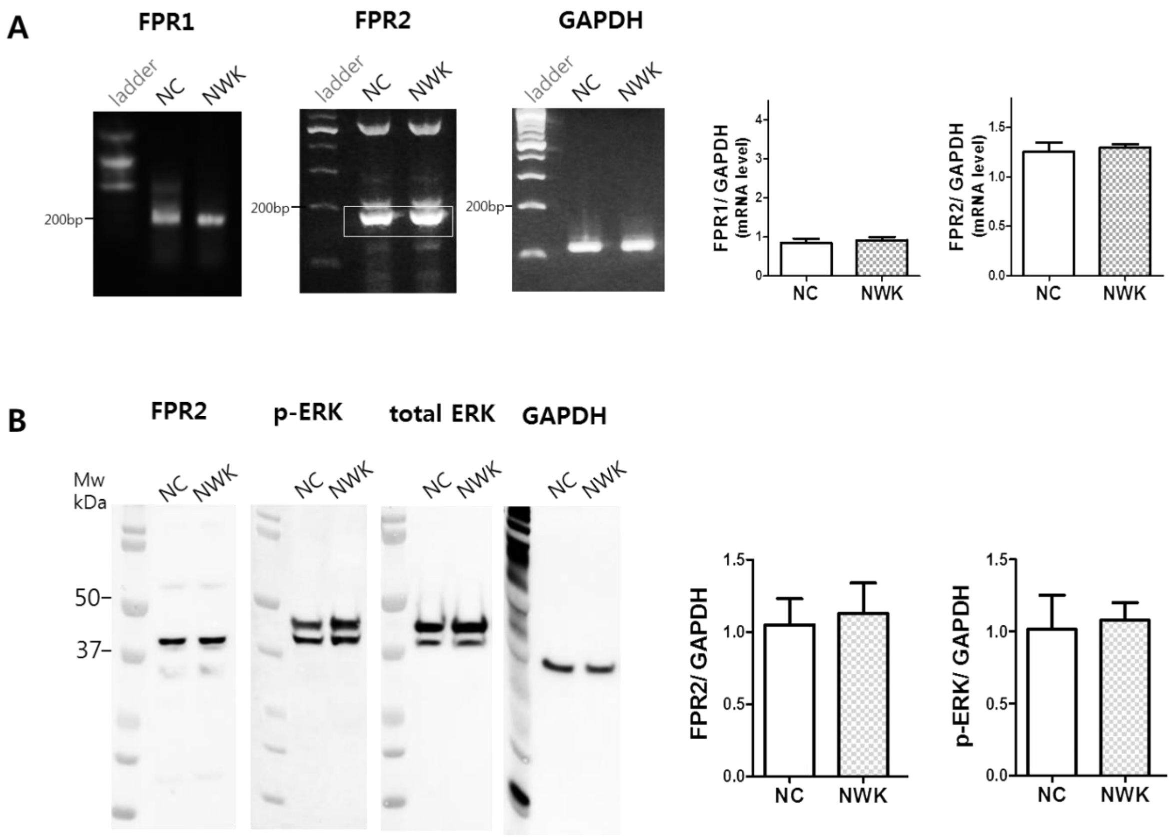
A
FPR1
FPR2
GAPDH
ladder
NC
NWK
200bp
FPR1/ GAPDH (mRNA level)
FPR2/ GAPDH (mRNA level)
B
FPR2
p-ERK
total ERK
GAPDH
Mw kDa
50
37
FPR2/ GAPDH
p-ERK/ GAPDH

Figure S5

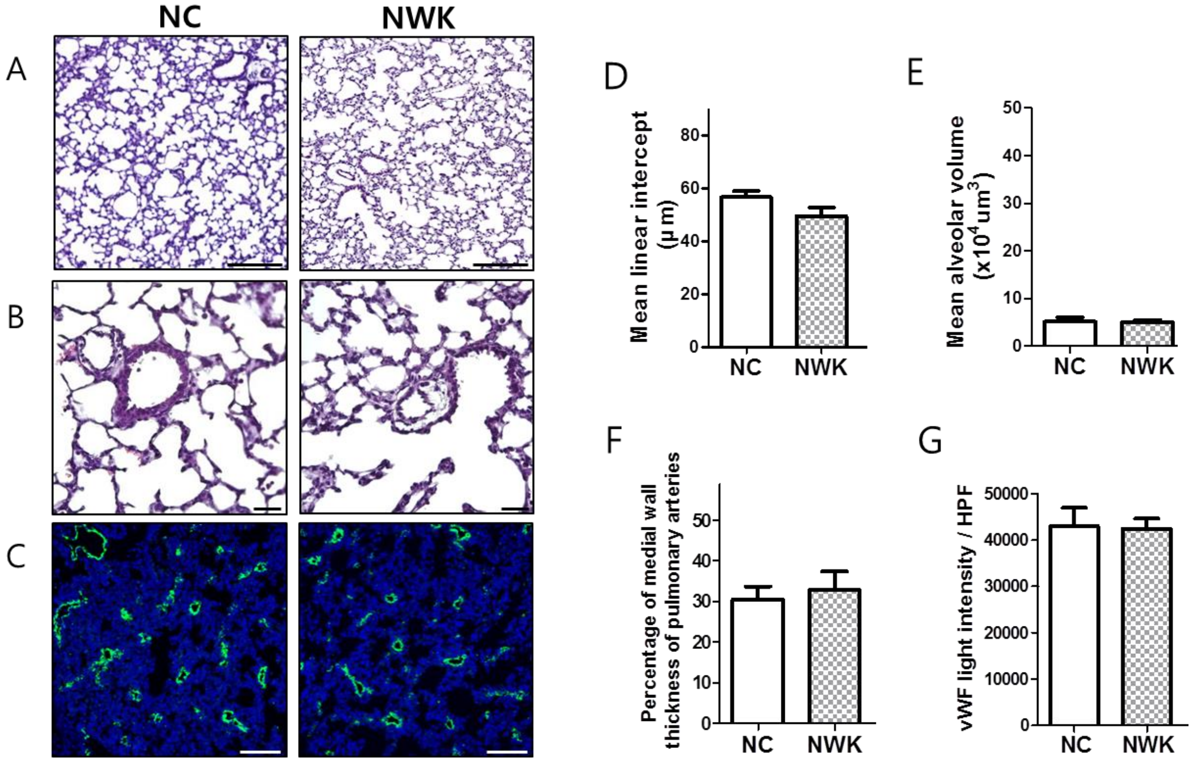

NC
NWK
A
B
C
D
Mean linear intercept (μm)
0
20
40
60
80
NC
NWK
E
Mean alveolar volume (x10⁴um³)
0
10
20
30
40
50
NC
NWK
F
Percentage of medial wall thickness of pulmonary arteries
0
10
20
30
40
50
NC
NWK
G
vWF light intensity / HPF
0
10000
20000
30000
40000
50000
NC
NWK

Figure S6

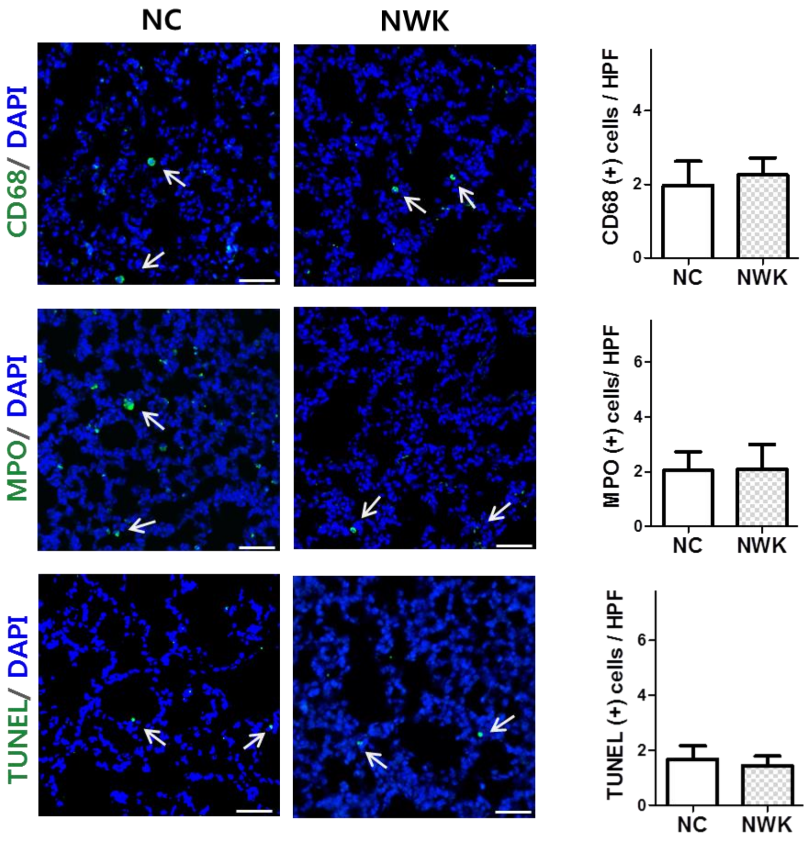

NC
NWK
CD68/ DAPI
MPO/ DAPI
TUNEL/ DAPI
CD68 (+) cells / HPF
MPO (+) cells/ HPF
TUNEL (+) cells / HPF
0
2
4
6
NC
NWK

Figure S7

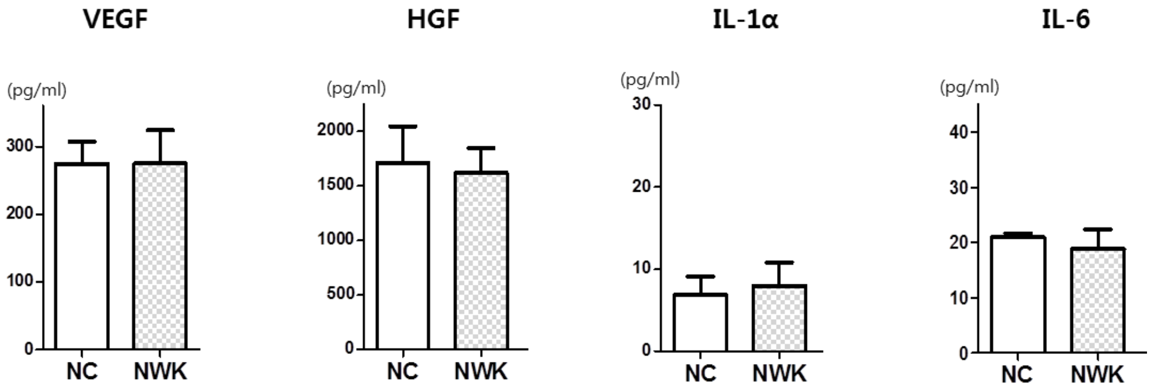
VEGF
(pg/ml)
300
200
100
0
NC
NWK
HGF
(pg/ml)
2000
1500
1000
500
0
NC
NWK
IL-1α
(pg/ml)
30
20
10
0
NC
NWK
IL-6
(pg/ml)
40
30
20
10
0
NC
NWK

Figure S8

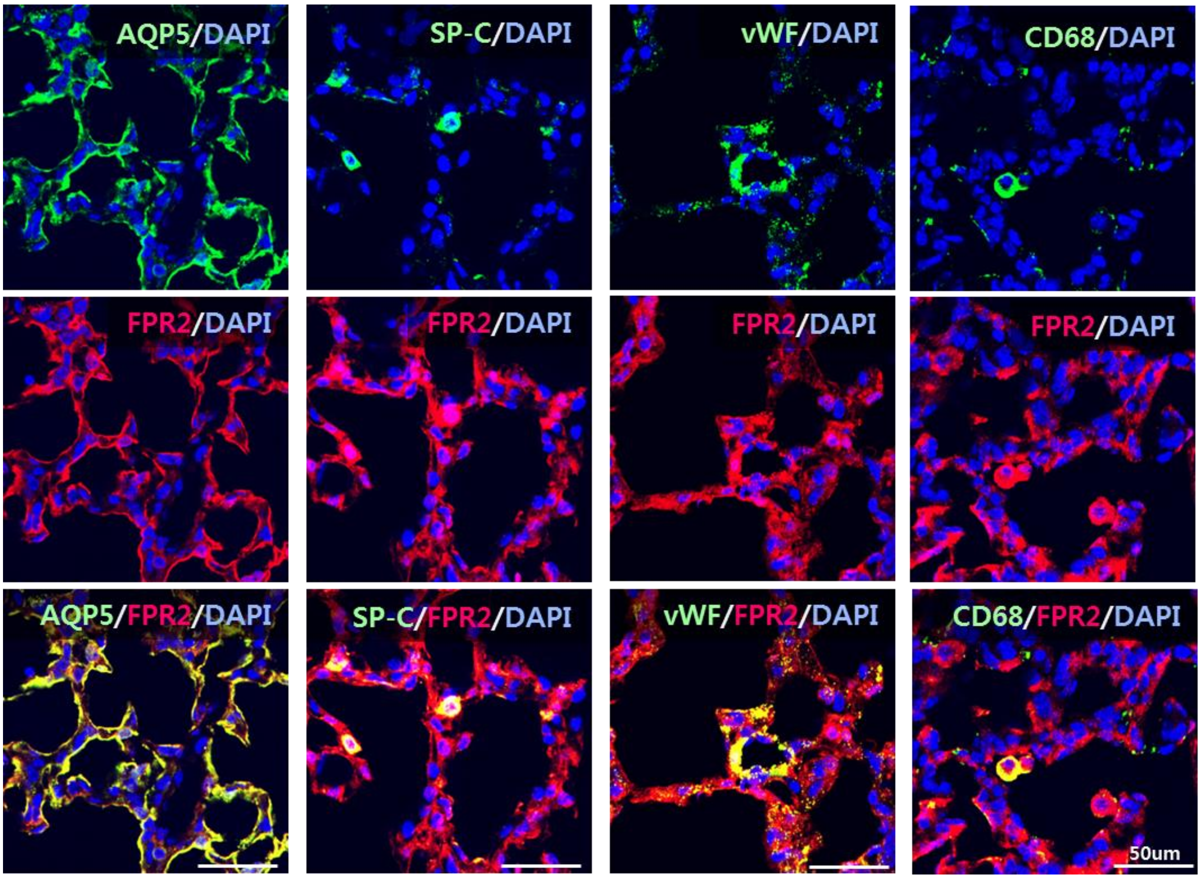
AQP5/DAPI
SP-C/DAPI
vWF/DAPI
CD68/DAPI
FPR2/DAPI
FPR2/DAPI
FPR2/DAPI
FPR2/DAPI
AQP5/FPR2/DAPI
SP-C/FPR2/DAPI
vWF/FPR2/DAPI
CD68/FPR2/DAPI
50um

Figure S9

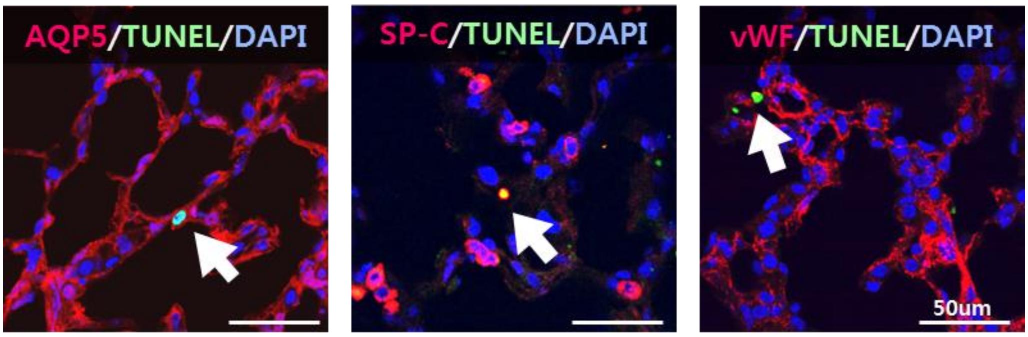

Supplement: Supplementary file 1 — supplementary material [file 41598_2019_43321_MOESM1_ESM.pdf]
